# 

**Published:** 1850-12

**Authors:** 


### CONTENTS

OF NO. VI.

### ORIGINAL COMMUNICATIONS.

### ESSAYS AND CASES.

Art. I.—On the History of Quinine, the preparations of that article, and their use in medicine. By Theodore S. Bell, M.D., of Louisville, Ky. - - - 461

Art. II.—Mercurials in the treatment of Typhoid Fever. By M. A. Becquerel. Translated from the *Comptes Rendus,* by D. W. Yandell, M.D., of Louisville, Ky. 509

### BIBLIOGRAPHICAL NOTICES.

Art. III.—The Principles and Practice of Dental Surgery. By Chapin A. Harris, M.D., D.D.S., Professor of the Principles and Practice of Dental Surgery in the Baltimore College; Fellow of the American Society of Dental Surgeons; Member of the American Medical Association, etc., etc. Fourth edition: revised, modified, and greatly enlarged. With two hundred Illustrations. - - - - - - - - - 516

Art. IV.—Observations on certain of the Diseases of young Children. By Charles D. Meigs, M. D., Professor of the Diseases of Women and Children in the Jefferson Medical College at Philadelphia, etc. etc. - - 519

Art. V.—Of the Causes, Nature, and Treatment of Palsy and Apoplexy: of the Forms, Seats, Complications, and Morbid Relations of Paralytic and Apoplectic Diseases. By James Copland, M.D., F.R.S., etc. etc. - 521

Art. VI.—Human Physiology. By Robley Dunglison, M. D., Professor of the Institutes of Medicine in Jefferson Medical College, Philadelphia. With nearly 500 illustrations. Seventh edition, thoroughly revised, and extensively modified and enlarged. In two volumes. - 523

Art. VII.—General Therapeutics and Materia Medica, adapted for a medical text book. By Robley Dunglison, M.D., etc. One hundred and eighty-two illustrations. Fourth edition, revised and improved. In two vols. - 525

Art. VIII.—Elementary Chemistry, theoretical and practical. By George Fownes, F.R.S., Professor of Practical Chemistry in University College, London. Edited, with additions, by Robert Bridges, M. D., Professor of Chemistry in the Philadelphia College of Pharmacy, etc., etc. Third American, from a late London edition. With numerous wood engravings. - - - - 526

Art. IX.—A Practical Handbook of Medical Chemistry. By J. E. Bowman, Demonstrator of Chemistry in King's College, London; and author of "Practical Chemistry." 528

Art. X.—Surgical Anatomy. By Joseph Maclise, Surgeon. With colored plates. Part third. - - - - - 530

Art. XI.—Spectacles: their Uses and Abuses in long and short sightedness; and the Pathological conditions resulting from their irrational employment. By J. Sichel, M.D., of the Faculties of Berlin and Paris; Clinical Professor of Diseases of the Eye; Officer of the Legion of Honor, etc. etc. etc. Translated from the French, by permission of the author, by Henry W. Williams, M.D., Fellow of the Massachusetts Medical Society. - 531

Art. XII.—The American Journal of Science and Arts.—Conducted by Professors B. Silliman, and B. Silliman, jr., and James D. Dana. No. 30. November, 1850. With Index to vols. I—X. - - - - 535

SELECTIONS AND SYNOPSIS.

Professor Austin Flint's Appeal. - - - - - - 538

Professor Eve's Card. - - - - - - - 541

The Use of Tobacco. - - - - - - - - 542

ORIGINAL INTELLIGENCE.

Close of Volume Sixth—Third Series. - - - - - 545

Registration. - - - - - - - - - - 547

Selected Matter - - - - - - - - 548

Index. - - - - - - - - - - - 549

